# Metabotropic Glutamate Receptor 5: A Potential Target for Neuropathic Pain Treatment

**DOI:** 10.2174/1570159X23666241011163035

**Published:** 2024-10-14

**Authors:** Chalton Manengu, Chun-Hao Zhu, Guo-Dong Zhang, Miao-Miao Tian, Xiao-Bing Lan, Li-Jun Tao, Lin Ma, Yue Liu, Jian-Qiang Yu, Ning Liu

**Affiliations:** 1 College of Pharmacy, Ningxia Medical University, 1160 Shengli Street, Yinchuan, 750004, Ningxia Hui Autonomous Region, China;; 2 School of International Education, Ningxia Medical University, 1160 Shengli Street, Yinchuan 750004, Ningxia Hui Autonomous Region, China;; 3 Department of Pharmacy, People’s Hospital of Ningxia Hui Autonomous Region, Yinchuan, 750004, China

**Keywords:** mGlu5, GPCRs, neuropathic pain, glutamate, ion channel, pain treatment

## Abstract

Neuropathic pain, a multifaceted and incapacitating disorder, impacts a significant number of individuals globally. Despite thorough investigation, the development of efficacious remedies for neuropathic pain continues to be a formidable task. Recent research has revealed the potential of metabotropic glutamate receptor 5 (mGlu5) as a target for managing neuropathic pain. mGlu5 is a receptor present in the central nervous system that has a vital function in regulating synaptic transmission and the excitability of neurons. This article seeks to investigate the importance of mGlu5 in neuropathic pain pathways, analyze the pharmacological approach of targeting mGlu5 for neuropathic pain treatment, and review the negative allosteric mGlu5 modulators used to target mGlu5. By comprehending the role of mGlu5 in neuropathic pain, we can discover innovative treatment approaches to ease the distress endured by persons afflicted with this incapacitating ailment.

## 1 INTRODUCTION

Neuropathic pain (NP) arises as a result of damage or illness affecting the somatosensory nerve system [1]. According to a 2017 article [2], this characteristic has been prevalent in the clinic for many years and has impacted approximately 7-10% of individuals. It is possible to feel skin burning or experience an electric shock. It can have a significant influence on overall well-being, resulting in persistent discomfort and restrictions in daily activities [3]. Three types of neuropathic pain exist: central, peripheral, or mixed. The impairment or malfunction of the brain or spinal cord causes central neuropathic pain, while damage or dysfunction of peripheral nerves causes peripheral neuropathic pain. Mixed neuropathic pain is characterized by the simultaneous involvement of both central and peripheral processes. The causes of peripheral neuropathic pain encompass peripheral nerve injury, chemotoxicity (resulting from chemotherapy), metabolic disorders (such as diabetes), and viral infections such as HIV and herpes zoster [4, 5]. The causes of central neuropathic pain might include traumatic events such as post-stroke pain, spinal cord injury, and multiple sclerosis [1, 6]. Managing neuropathic pain can be difficult, and conventional analgesics may not offer sufficient pain relief. Therefore, there is a need for new research studies focused on finding innovative drugs that can effectively alleviate intense pain and improve the overall well-being of patients.

After a nerve injury, the neurological system may process and transmit pain signals differently [7]. These changes may be permanent or temporary. The brain processes pain signals, which constitute the actual feeling of pain. When a nerve sustains damage, the brain struggles to accurately interpret the signals originating from the body's painful sensations. This could result in the development of chronic pain syndromes, decreased pain thresholds, and increased pain sensitivity. According to reports, damage to the nerves can also trigger an inflammatory reaction in the nervous system, with the microglial (non-neuronal) cells playing a critical role in this process [8]. The microglia can initiate neuroinflammatory responses that may contribute to the development and maintenance of chronic pain [9]. Because inflammation affects the nervous system, it is possible that pain signals will become more intense. Nerve damage is one of the things that might set off a process known as neural plasticity [10], which refers to the potential of the nervous system to change and adapt in response to injuries or other stimuli. These modifications can make the neurological system more sensitive to pain, which can then contribute to the development of chronic pain syndromes.

### 1.1 Peripheral Mechanism in Neuropathic Pain

Chronic neuropathic pain can arise from peripheral nerve injury through several mechanisms. While the initial damage may be localized, the resulting chronic neuropathic pain can extend beyond the affected location and impact other parts of the body. Peripheral sensitization occurs when injured cells release inflammatory mediators, leading to an amplified reaction. Furthermore, sympathetic neuron growth in the dorsal root ganglia has increased [11]. Toxins, drugs like vincristine and paclitaxel, metabolic damage, and cytokines all have severe effects on the ends of pain-processing C and A fibers that are not myelinated [12]. This increases the likelihood of experiencing neuropathic pain, which can lead to alterations in fiber density and heightened neuronal excitability. Aberrations in the axons lead to fiber deterioration and changes in channel expression and composition, which can cause abnormal firing and inaccurate transmission of signals [13, 14]. These injuries and modifications to the peripheral nerve result in neuropathic pain.

### 1.2 Central Mechanism in Neuropathic Pain

Central sensitization (CS) refers to the heightened responsiveness of nociceptive neurons in the central nervous system (CNS) to their incoming sensory signals that are below the threshold for activation [15, 16]. CS plays a role in affecting pain facilitation and inhibition, inhibiting descending pathways [17] and over-activation of ascending and pain facilitatory pathways [18]. The persistent input from peripheral C fibers leads to heightened excitability of the spinal cord (Table **1**). Following an increased responsiveness of nociceptive neurons, alterations occur in the synapses of neurons, including changes in synapse location and calcium ion permeability. These modifications contribute to the development of persistent discomfort [19, 20]. The spinal and supraspinal nociceptive pathways exhibit heightened sensitivity in response to a stimulus [21, 22]. An equilibrium change between the descending facilitation and inhibition in the supraspinal pathways contributes to chronic pain [12].

## 1.3 Treatment of Neuropathic Pain

The degree to which treatments for neuropathic pain are successful can vary from patient to patient. Finding the treatment that works best for a particular patient, or the treatment that works best in combination with other treatments, may require some trial and error. It is highly recommended that an individualized treatment strategy be designed in close collaboration with a qualified medical practitioner. Neuropathic pain stems from abnormalities in pain signaling, although the precise causes of this type of pain remain unidentified [23, 24]. It is possible that treating neuropathic pain will be challenging due to the complexity of the condition. In multimodal methods for neuropathic pain therapy, it is common practice to combine a number of different treatments in order to achieve the best possible outcomes. Medications are often the first line of treatment for neuropathic pain [25]. The available drugs used to manage neuropathic pain include antipsychotics, anticonvulsants, and antidepressants [26] (Table **2**).

### 1.3.1 Antidepressants

In the treatment of neuropathic pain, serotonin-norepinephrine reuptake inhibitors (SNRIs) and tricyclic antidepressants (TCAs) have demonstrated analgesic efficacy. According to several sources, SNRIs and TCAs are both effective first-line medications for neuropathic pain [25, 27-32]. The efficacy of antidepressants in alleviating neuropathic pain is dependent upon the particular antidepressant used. Based on clinical research, TCAs are more effective in relieving pain compared to placebos. TCAs show that the Number-Needed-To-Treat (NNTs) is 3.6, while SNRIs have an NNT of 6.4 for 50% pain relief [33]. TCAs commonly used for the treatment of NP include amitriptyline and nortriptyline [26]. These treatments have been proven to be beneficial in several types of neuropathic pain, including peripheral neuropathy and post-herpetic neuralgia [31]. Although amitriptyline is known to inhibit sodium channel activity [34], most TCAs have been found to exert their effects by decreasing the reuptake of serotonin and noradrenaline [35]. These mechanisms help relieve pain sensations. However, because of side effects like drowsiness, vertigo, dry mouth, and urine retention, their use in treating neuropathic pain may be limited [36]. In addition, peripheral diabetic neuropathy, severe peripheral neuropathy, and central neuropathic pain are also frequently treated with SNRIs, such as venlafaxine and duloxetine [25, 31]. SNRIs work by blocking the reuptake of norepinephrine and serotonin, which increases levels of neurotransmitters implicated in pain modulation [29, 30]. Analgesic effects of selective serotonin-norepinephrine reuptake inhibitors (such as duloxetine) have been reported [37].

### 1.3.2 Anticonvulsants

Gabapentin and pregabalin, along with other anticonvulsant drugs, are commonly prescribed as the first choice of treatment for treating neuropathic pain [28, 31, 38]. The effectiveness of anticonvulsants in neuropathic pain relief is often measured by the NNT to yield 30-50% pain relief [39]. These drugs function by stabilizing overactive nerve cells, hence decreasing pain signals. Gabapentin and pregabalin attach to the alpha-2/delta-1 subunit of voltage-gated calcium channels, which prevents the release of neurotransmitters and reduces central sensitization. Studies have demonstrated the efficacy of both gabapentin and pregabalin in treating post-herpetic neuralgia (PHN) [40, 41] and diabetic peripheral neuropathy (DPN) [38, 42]. The most frequent negative symptoms were drowsiness, fatigue, and swelling in the lower limbs [43]. Other anticonvulsants used for neuropathic pain include topiramate, carbamazepine, lamotrigine, lacosamide and phenytoin [44, 45]. The indication for topiramate is nerve pain, specifically postherpetic neuralgia [46].

Topiramate inhibits the Sodium/calcium channel to increase gamma-aminobutyric acid (GABA) and decrease alpha-amino-3-hydroxy-5-methyl-4-isoxazolepropionic acid (AMPA)-type glutamate receptors. Adverse effects associated with topiramate include cognitive impairment, hyponatremia, weight loss, and metabolic acidosis. While carbamazepine is indicated for trigeminal neuralgia [47]. Inactivated carbamazepine binds to voltage-dependent sodium channels to prolong the inactivated phase and block the transmission of action potentials.

### 1.3.3 Topical Medications

A number of studies have revealed that capsaicin and lidocaine patches are the topical treatments recommended for the treatment of neuropathic pain [48]. Applying topical medicines like lidocaine (5%) patches or capsaicin (8%) cream can achieve localized pain management. These ointments function by numbing the targeted area or reducing the sensitivity of the nerves. Applying a 5% lidocaine-medicated patch has proven to be quite efficient in treating PHN and DPN. It is frequently used as the initial treatment for postherpetic neuralgia, particularly in elderly people. Lidocaine blocks ectopic discharges of peripheral sodium channels [49, 50]. The literature currently available on topical therapy for peripheral neuropathic pain suggests that this kind of treatment may provide beneficial pain relief without the systemic adverse effects that are frequently associated with oral medicines [51]. The stated average success rate for topical treatment is 50% [52]. A study found that the NNT for capsaicin was 7.0 and 8.8 for pain alleviation [51]. Lidocaine patches have been suggested as a secondary treatment [30] and have been examined for their efficacy in alleviating peripheral neuropathic pain, specifically PHN. Nevertheless, the therapeutic advantage is negligible. Capsaicin has been proven to be effective in treating both diabetic and non-diabetic neuropathies [53, 54]. Capsaicin induces depolarization of the epidermal nerve fibers. It usually causes depolarization and desensitization of nerve fibers in the skin by activating the transient receptor potential vanilloid 1 (TRPV1) present on Aδ and C-nerve fibers. The nerve depolarization triggers the secretion of substance P. Extended exposure to capsaicin depletes substance P, resulting in nerve desensitization and the reversal of nerve degeneration [55]. However, due to the unpleasantness of applying it (Table **2**) and the need for frequent use, many people find it challenging to adhere to a capsaicin regimen [56]. Capsaicin, at a concentration of 8%, may be suggested as a therapy option for neuropathic pain when other treatments have not been effective [56-60].

### 1.3.4 Other Drugs

In addition, weak opioids such as tramadol have been widely used in the management of peripheral neuropathic pain because of their ability to inhibit the reuptake of serotonin and norepinephrine. However, experts recommend using tramadol as a third-line drug for treatment, cautioning against its addictive nature and susceptibility to abuse [28, 25]. Research has demonstrated the efficacy of tramadol in the management of diabetic neuropathy [61], postherpetic neuralgia [62], and neuropathic pain associated with cancer [63]. Other drugs that have been reported to be of use in neuropathic pain include tapentadol [64]. Tapentadol is a norepinephrine reuptake inhibitor and a modest µ-receptor agonist. Its greater potency compared to tramadol makes it a third- or fourth-line therapy [30]. Some studies have indicated that tapentadol is effective in treating diabetic neuropathy [65].

In summary, despite the availability of various pharmacological treatment options, many patients suffering from NP still experience inadequate pain relief or intolerable side effects. Therefore, there is a need to investigate alternative targets for the management of NP, such as the metabotropic glutamate receptor 5 (mGlu5). mGlu5 is a promising target for NP because it plays a crucial role in modulating pain signaling in the CNS. One of the key advantages of targeting mGlu5 in NP treatment is its expression in various regions of the brain and spinal cord, which are known to be important in the processing of pain signals. Studies have shown that activation of mGlu5 increases the release of neurotransmitters involved in pain transmission and modulation. Therefore, targeting mGlu5 may provide a more targeted approach to modulating pain signaling and potentially reduce the side effects associated with current pharmacological treatments for NP.

## 2 METABOTROPIC GLUTAMATE RECEPTOR

Glutamate is the major excitatory neurotransmitter in the CNS. Glutamate regulates cell excitability and synaptic transmission through second messenger signaling pathways; this is mediated by glutamate receptors called metabotropic glutamate receptors (mGlus) and ionotropic glutamate receptors (iGlus) [66]. iGlus are ligand-gated ion channels that mediate excitatory synaptic transmission in the CNS and are key players in synaptic plasticity. There are three subtypes of iGlus based on their ligand binding properties and sequence similarity: AMPA receptors, kainate receptors, and N-methyl-D-aspartate (NMDA) receptors [67]. While mGlus belongs to the G-protein coupled receptor (GPCR) superfamily. GPCRs are transmembrane proteins that are stimulated by ligands such as neurotransmitters. They then convey signals within the cell by interacting with G-proteins. mGlus can be subdivided into three groups based on their homology; these include Group I, Group II, and Group III [68]. For the most part, group I metabotropic receptors like mGlu1 and mGlu5 are connected with stimulatory processes, including phospholipase C activation and the synthesis of second messengers like inositol and diacylglycerol. The mGlu2 and mGlu3 receptors are classified as Group II, whereas the mGlu4, mGlu6, mGlu7, and mGlu8 receptors are assigned to Group III. Group III receptors, like Group II receptors, inhibit glutamatergic neurotransmission [69]. They are also pre-synaptically expressed to modulate non-glutamatergic neurons [70, 71].

The expression and functionality of group I metabotropic glutamate receptors are limited to glial cells, presynaptic terminals, and postsynaptic terminals. The majority of group I metabotropic glutamate receptors are located in the postsynaptic regions situated at the periphery. Activation of Group I metabotropic glutamate receptors leads to many postsynaptic effects, including depolarization, stimulation, and spike frequency adaptation, in multiple regions of the brain [72]. The central nervous system has an abundance of group II mGlu (mGlu2/3) [73-75]. mGlu2 is often found on the presynaptic terminals of the periphery, but mGlu3 has a more diversified distribution, being heavily concentrated on glial cells [70, 76]. On the other hand, Group III mGlus are mainly found in presynaptic active regions, especially at the axon terminal. Reduced synaptic transmission of glutamatergic or GABAergic impulses is the result of the inhibition of neurotransmitter release by group III mGlus [77]. This article focuses on mGlu5 as a potential target for neuropathic pain treatment.

### 2.1 Metabotropic Glutamate Receptor 5 (mGlu5)

Metabotropic Glutamate Receptor 5 (mGlu5) is a member of group I metabotropic receptors. There are two recognized splice variants of mGlu5, referred to as mGlu5a and mGlu5b. mGlu5b has more amino acid residues compared to the isoform mGlu5a, which lacks an additional sequence. The diverse forms of mGlu5 have been discovered in various areas of both the human and adult rat brain [78, 79]. And mGlu5b is more abundant than mGlu5a in the adult spinal cord dorsal horn. These splice variants produce Cl^-^ current that is activated by Ca^2+^ and controls the growth of neurites. mGlu5a inhibits the formation and development of neurites, while mGlu5b promotes the growth of neurites. In addition, mGlu5a and mGlu5b stimulate the synthesis of inositol phosphate (IP) and cyclic adenosine monophosphate (cAMP). Both variations exhibit enhanced basal phospholipase C (PLC) activity, suggesting intrinsic interaction activity with homer proteins. Pharmacologically, both mGlu5a and mGlu5b exhibit identical properties [80].

The activities of mGlu5 are primarily excitation-based. It is highly expressed in neurons located in the dorsal horn of the spinal cord [81]. Expression levels of mGlu5 have also been observed in the cerebral cortex, hippocampus, subiculum, nucleus accumbens, striatum, olfactory bulb, and lateral septal nucleus [82, 83]. The mGlu5 receptor in the dorsal horn of the spinal cord is crucial in the modulation of pain-related processes, such as increased sensitivity to pain following nerve injury, through the influence of glutamate-induced plasticity. Through its interactions with the NMDA receptor, mGlu5 promotes neural plasticity. According to earlier research, GABA's effects are enhanced when mGlu5 receptors are activated, especially in the nucleus [84]. Consequently, it has been suggested that GABA-related excitatory and inhibitory signaling pathways can be controlled by activating metabotropic glutamate receptors. Pre-synaptic mGlu5 receptors are involved in controlling neuronal excitability and synaptic plasticity to preserve homeostasis [85]. In addition, activation of mGlu5 in astrocytes can have an effect on neuroinflammatory processes [86]. The identification and characterization of mGlu5 paved the way for additional research into both its physiological and pathological activities, including the involvement of neuropathic pain development. According to a study by Vincent *et al*., mGlu5 may play a critical role in the onset of neuropathic pain [87]. They showed that mGlu5 expression in the spinal cord dorsal horn significantly increased after spared nerve injury to induce neuropathic pain, and blocking mGlu5 attenuated painful behaviors.

### 2.2 Structure of Group I Metabotropic Glutamate Receptors

The structure of group I metabotropic glutamate receptors (mGlu) is defined by the presence of a number of essential components. Both mGlu1 and mGlu5 are members of the GPCRs subclass that is referred to as group I mGlus. They are composed of a single polypeptide chain that is comprised of seven different membrane-spanning sections that are referred to as seven transmembrane (7TM) domains [88]. These receptors are engaged in a wide variety of physiological activities in the brain and nervous system, such as pain perception [89]. These transmembrane domains are connected by six loops total: three loops inside the cell and three loops outside the cell.

### 2.3 Ligand Binding Domains

The extracellular regions of group I mGlus contain two separate domains that are important in the process of ligand binding [90, 91]. These domains are referred to as the Venus flytrap domain (VFT) and the cysteine-rich domain (CRD). Glutamate is the principal ligand for mGlus, and the VFT domain, which is positioned at the N-terminus, is the region responsible for its binding. The CRD is positioned between the VFT domain and the transmembrane domain. The primary role of the CRD is to interconnect the VFT domain to the transmembrane domain [91]. Though it is not clear what role CRD really plays in the binding of ligands to mGlus, some studies have reported that ligand binding induces signal transmission from the VFT domain through the CRD to mGlu [90]. The CRD has the potential to shift toward the transmembrane domain in the glutamate-binding region. This proximity can be attributed to the rearrangement and closure of the bilobed VFTs, which is a consequence of glutamate binding. The CRD is then able to transmit signals from VFTs to the transmembrane domain [92].

The full-length cryo-electron microscopy (EM) and biophysical studies of the orthosteric binding site contribute to an understanding of the receptor activation and ligand binding mechanisms for mGlu5. The dimer structure of full-length mGlu5 with bound Nb43 in the absence of an orthosteric ligand is identical to the apo-state, as shown by cryo-EM analysis [93, 94]. This suggests that Nb binding alone does not close the VFTs but rather limits their opening to an entirely inactive conformation. Using a mix of cryo-EM, X-ray crystallography, and biochemical assays, Koehl *et al*. (2019) obtained structures of the mGlu5 dimer in both its active and inactive states to understand how agonist binding at the VFT is relayed to the intracellular G protein-coupling region of the 7TM domain. This strategy aimed to enhance receptor stability by combining orthosteric and allosteric small molecule ligands with a nanobody, namely a single-chain camelid antibody [94]. Additionally, the cryo-EM displays the receptor's whole 7TM domains, as well as its N-terminal domain, peptide ligand, and signal transduction G protein [95].

The ligand binding site of mGlu5 denotes the distinct site on the receptor where ligands, including glutamate or other small molecules, can attach and engage in interaction with the receptor. The initiation of the signaling cascade within the cell occurs as a result of ligand binding to the ligand binding site. The distinct ligand binding sites of metabotropic glutamate receptor 5 are:

#### 2.3.1 Orthosteric Ligand Binding Pocket

mGlu5 exhibits a characteristic behavior of forming obligatory dimers, wherein two subunits of the receptor combine to generate a functioning receptor complex consisting of an orthosteric ligand binding site in the VFT domain [95]. The VFT domain of the receptor's extracellular region contains the binding pocket. The VFT domain is comprised of two lobes that experience conformational alterations following the interaction of a ligand. The binding of glutamate to the orthosteric binding pocket occurs through the interaction between glutamate and particular amino acid residues located within the pocket.

#### 2.3.2 Allosteric Ligand Binding Pocket

**Fig. (1):**
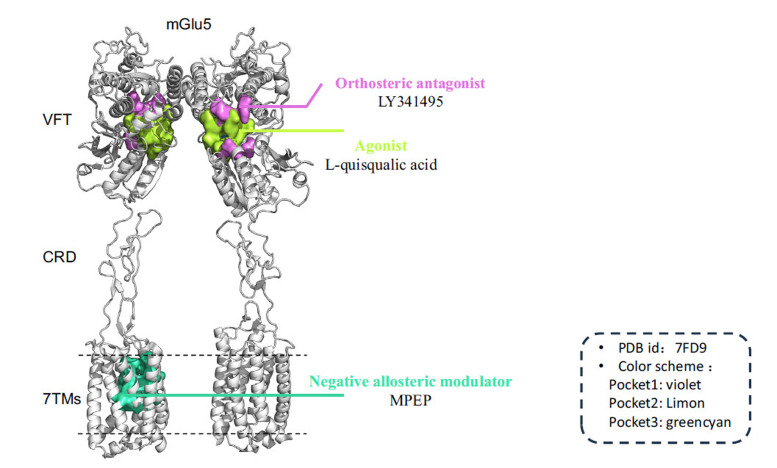
Crystal structure of metabotropic glutamate receptor 5 (PDB code 7FD9). The diagram displays the dimeric form of mGlu5, with three different compounds highlighted in three distinct colors, each binding to a pocket on mGlu5 (Pocket1: violet, Pocket2: Limon, Pocket3: green cyan) [93]. (The structure was processed and optimized by biovia discovery studio and Pymol).

Allosteric ligands exhibit the ability to bind to locations that are separate and distinct from the orthosteric binding site. As a result, allosteric modulators are able to control the GPCR’s signal transduction pathways and their conformational change [96]. Allosteric modulators of GPCRs have distinct pharmacological effects on receptor signaling. They can be classified into two categories: (i) positive allosteric modulators (PAMs), which work together with orthosteric agonists to amplify downstream signals, and (ii) Negative allosteric modulators (NAMs) are a type of allosteric modulator that attenuate the pharmacological actions of an orthosteric ligand that is attached to the receptor [97-99]. On the other hand, there are allosteric modulators (ago-PAMs) that are effective without the orthosteric ligand. These allosteric modulators display the inherent effectiveness of orthosteric and allosteric ligands [100] (Fig. **1**).

## 3 METABOTROPIC GLUTAMATE RECEPTOR 5 AND NEUROPATHIC PAIN

There is abundant expression of mGlu5 in different regions of the brain, including the primary somatosensory (S1) cortex. Active neural networks in the S1 cortex process crucial somatosensory signals, such as the intensity of pain. Manipulating the S1 cortex changes the way the thalamus and anterior cingulate cortex process pain transmissions in the brain. The S1 cortex might play a role in the development and persistence of neuropathic pain [101, 102]. According to Danjo *et al*. (2022), neuropathic pain induction was facilitated by S1 astrocytic mGlu5, after partial sciatic nerve ligation, the astrocytes in the S1 cortex were crucial in triggering mechanical allodynia [103]. In addition, astrocytes go through central sensitization; they transform into “reactive astrocytes”. Reactive astrocytes release a variety of signaling molecules, including glutamate, ATP, and cytokines. In addition to increasing pain sensitivity, these signaling molecules can control neuronal excitability. The ability of astrocyte-expressed mGlu5 to release glutamate has the potential to enhance neuronal excitability [104].

In astrocytes, mGlu5 promotes the expression of purinergic receptor P2X3, potentially leading to the development of neuropathic pain [105]. P2X3 is a non-selective ligand-gated ion channel. It is found in astrocytes in the spinal cord [106]. Nerve damage releases adenosine triphosphate (ATP), which activates the presynaptic membrane's P2X3 receptor and triggers Ca^2+^ influx, phosphorylating PKA and PKC and releasing glutamate. P2X3 activation in the DRG promotes aberrant nerve discharge and visceral hyperalgesia [107, 108]. A study by Mah *et al*. (2017) found that chronic constriction injury of infraorbital nerve (CCI-ION) rat model elevated astrocytic P2X3 expression, which resulted in the development of mechanical allodynia [105]. Nevertheless, the administration of MPEP reduced the intensity of this mechanical allodynia. Astrocytic P2X3 overexpression after CCI-ION may be associated with the activation of astrocytic mGlu5 in response to glutamate release from astrocytes after nerve damage. In astrocytes, mGlu5 activation promotes ERK2 phosphorylation [109, 110], which is involved in neuropathic pain, and inhibiting ERK2 activation may downregulate P2X3 expression [111]. These findings support the hypothesis that activation of mGlu5 indirectly interacts with P2X3, leading to the development of neuropathic pain. Nevertheless, further research is required to elucidate the precise mechanisms implicated in this interaction.

**Fig. (2):**
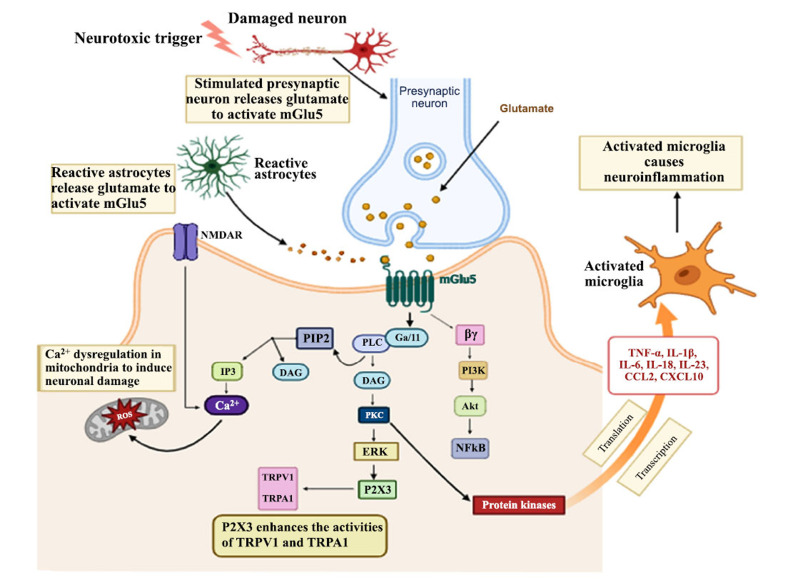
Schematic illustration of the role of mGlu5 activation in neuropathic pain. Activation of mGlu5 stimulates the PLC, producing IP3 and diacylglycerol. IP3 releases calcium from cellular stores, activating calcium-dependent ion channels; intracellular calcium and DAG stimulate PKC and its associated downstream signaling pathways. Activation of mGlu5 also activates the PI3K signaling pathway with the subsequent activation by phosphorylation of the pro-survival protein kinase Akt [created in BioRender].

Activation of mGlu5 causes pain-related behaviors. In a study to demonstrate that mGlu5 activation played a role in pain generation, rats were subjected to a Periphery. Intraplantar (i.p.) injection of a group I mGlu agonist (DHPG) or an mGlu5 agonist (CHPG) resulted in mechanical and thermal hyperalgesia. These sensations were later suppressed in a dose-dependent manner by a microinjection of mGlu5 NAM (MPEP), indicating that mGlu5 activation causes pain [112]. Furthermore, there have been reports of nociceptive behaviors induced by spinal mGlu5 activation as well. Research has shown that certain behaviors, like cold hypersensitivity, can be induced by group I mGlu agonists like DHPG or 1S,3R-ACPD when administered intrathecally in the spinal cord [113]. However, these behaviors associated with the activation of extracellular signal-regulated kinases ERK1 and ERK2 in the spinal cord can be and prevented by pretreatment with mGlu5 NAM [114]. Therefore, it can be concluded that nociceptive responses are mediated by activation of spinal mGlu5 (Fig. **2**).

The encoding and processing of pain signals is referred to as nociception, and it has been established that activation of mGlu5 is involved in the activation and modulation of nociceptive transmission [115], which contributes to inflammatory hyperalgesia [116]. mGlu5 receptors are expressed in pathways that are responsible for nociceptive sensations. There are a number of different channels through which mGlu5 activation might influence the transmission of nociceptive signals and susceptibility to pain. According to research, mGlu5 in the amygdala, ventrobasal thalamus, periaqueductal gray (PAG), and rostral ventromedial medulla has been associated with modifying nociceptive processing [117-119]. A study by Chung *et al*. (2020) found that mGlu5 in the PAG region is constantly active and helps regulate neuronal excitability, which is important for pain regulation. If the activities of mGlu5 in the PAG area are reduced, it may lead to increased pain sensitivity [120]. Activation of mGlu5 in the Ventrobasal thalamus amplifies neuronal reactions to painful stimuli. On the other hand, blocking mGlu5 decreases the neuronal reactions [121]. Furthermore, Pain affects the amygdala's basolateral (BLA) and central nucleus (CeA). Electrically manipulating CeA activity does not change spontaneous nociceptive behaviors [122, 123], but chronic pain increases CeA synaptic transmission, which induces or maintains hypersensitivity. In addition, mGlu5 excites neurons in the CeA when the visceral is stimulated [124, 125]. The mGlu5 then activates extracellular signal-regulated kinases 1/2 (ERK1/2), which may be crucial for painful response modulation [69, 126].

mGlu5 receptors may contribute to the initiation of nociceptive sensitization. Overall, preclinical models of inflammatory and neuropathic pain have shown pain-relieving effects when mGlu5 receptors were pharmacologically inhibited [127, 128]. Recent research has explored new photo-pharmacological techniques for controlling pain [129]. A study by Notartomaso *et al*. (2024) used photopharmacology to shed light on the involvement of mGlu5 in neuropathic pain. Photopharmacology is a novel method that uses light and photo-responsive chemicals to accurately regulate medication activity. They integrated optical manipulation techniques with the systemic administration of photo-switchable or photo-caged compounds in specific brain areas. They analyzed two light-sensitive mGlu5 ligands with distinct functions: compound JF-NP-26, a caged derivative of the mGlu5 receptor NAM raseglurant, which is inactive at room temperature but becomes active when exposed to light in the visible spectrum (405 nm); and photo-switchable mGlu5 receptor NAM alloswitch-1, which is active at room temperature but becomes inactive when exposed to light at 405 nm and can be re-activated by light at 520 nm. In a mouse model of cancer pain, the stimulation of JF-NP-26 in the thalamus by light resulted in rapid and potent pain relief [130].

There have also been reports of spinal cord involvement in cases of neuropathic pain. Several studies have reported an upregulation of activated mGlu5 in the spinal cord [113, 131, 132]. According to the findings of a study that was carried out by Kartha *et al*. (2021), there is a correlation between increased expression of spinal mGlu5 and the incidence of pain and spinal neuronal hyperexcitability later on following nerve root injury [133]. On the other hand, Hsieh *et al*. (2019) discovered that nerve injury resulted in a reduction in the expression of Hes1 and an increase in the transcription and expression of mGlu5 in the dorsal horn of the spinal cord. The interaction between Hes1, CDK9, and mGlu5 in the dorsal horn of the spinal cord had the effect of influencing the activity of RNA polymerase II and the expression of genes, which ultimately resulted in the development of neuropathic allodynia. When Hes1 levels were raised, the expression of mGlu5 was decreased, which resulted in less severe neuropathic pain [134]. Furthermore, Li *et al*. (2010) found that the activation of mGlu5 has a function in the enhancement of glutamatergic synaptic transmission in the spinal dorsal horn. This phenomenon is closely linked to the heightened excitability of neurons reported in neuropathic pain conditions. In addition, the researchers demonstrated that the activation of mGlu5 is a factor that contributes to a stimulus of aberrant firing and increased excitability in neurons that are located in the dorsal root ganglion (DRG) [131]. This discovery provides more evidence that mGlu5 plays a significant role in the increased excitability of neurons that are linked with neuropathic pain.

Further research has focused on mGlu5 and TRPV1 [135, 136]. TRPV1 is a non-selective cation channel mostly located in sensory neurons and plays a role in their function. It is referred to as the “heat and capsaicin receptor” due to its role in processing painful and uncomfortable stimuli. Inflammatory mediators can enhance TRPV1 sensitivity to pain, leading to heightened pain sensitivity. These receptors have been found to interact and impact the patient's perception of pain. There is evidence indicating a membrane-delimited relation between mGlu5 and the transient receptor potential vanilloid 1 channel in nociceptive sensory neurons [137]. By regulating TRPV1, mGlu5 can influence nociceptive signaling further. Peripheral mGlu5 modulates the activity of P2X3 receptors (as described above), which later enhances TRPV1 and TRPA1 activity during neuropathic pain [138]. Therefore, mGlu5 is indirectly involved in the stimulation of TRPV1, which contributes to the initiation and persistence of neuropathic pain.

### 3.1 Activation of Metabotropic Glutamate 5 Receptor in Neuropathic Pain

Metabotropic glutamate 5 receptor activation in pain is a description of how mGlu5 is triggered in response to painful stimuli. When activated, the effect of mGlus on cell excitability is expressed differently in various locations [139]. mGlus can be found in both presynaptic and postsynaptic neurons. Their presence has also been observed at peripheral nerve ends, glial cells, in the spinal cord, and supraspinal sites [140]. The mGlu receptors regulate the amplification of pain sensitivity in glial cells [141, 142]. Activation of group I mGlus has been documented to have the ability to either trigger or suppress the sensation of pain. Multiple studies have linked the activation of mGu5 receptors to many painful conditions, such as neuropathic pain [69, 112, 115, 143, 144]. Elevated levels of mGlu5 receptors have been detected in cases of spinal nerve damage and sciatic nerve ligation, leading to the development of heightened sensitivity to mechanical and thermal pain, as well as the experience of pain from normally non-painful stimuli (tactile allodynia) [145]. The study conducted by Urban *et al*. (2003) examined how the mGlu5 contributes to the persistence of heightened cold sensitivity in the chronic constriction injury (CCI) model [146]. The researchers stated that mGlu5 played a role in the development of cold hypersensitivity caused by CCI. Similarly, Zhu *et al*. (2004) investigated the involvement of the mGlu5 receptor in various pain models, such as neuropathic pain, visceral pain, postoperative pain, and inflammatory pain. According to their findings, MPEP, an mGlu5 antagonist, effectively reduced the intensity of heightened sensitivity to thermal hyperalgesia in a model of neuropathic pain caused by spinal nerve ligation [147]. However, it had only a slight effect on the reduced sensitivity to mechanical allodynia at a lower dose.

**Fig. (3):**
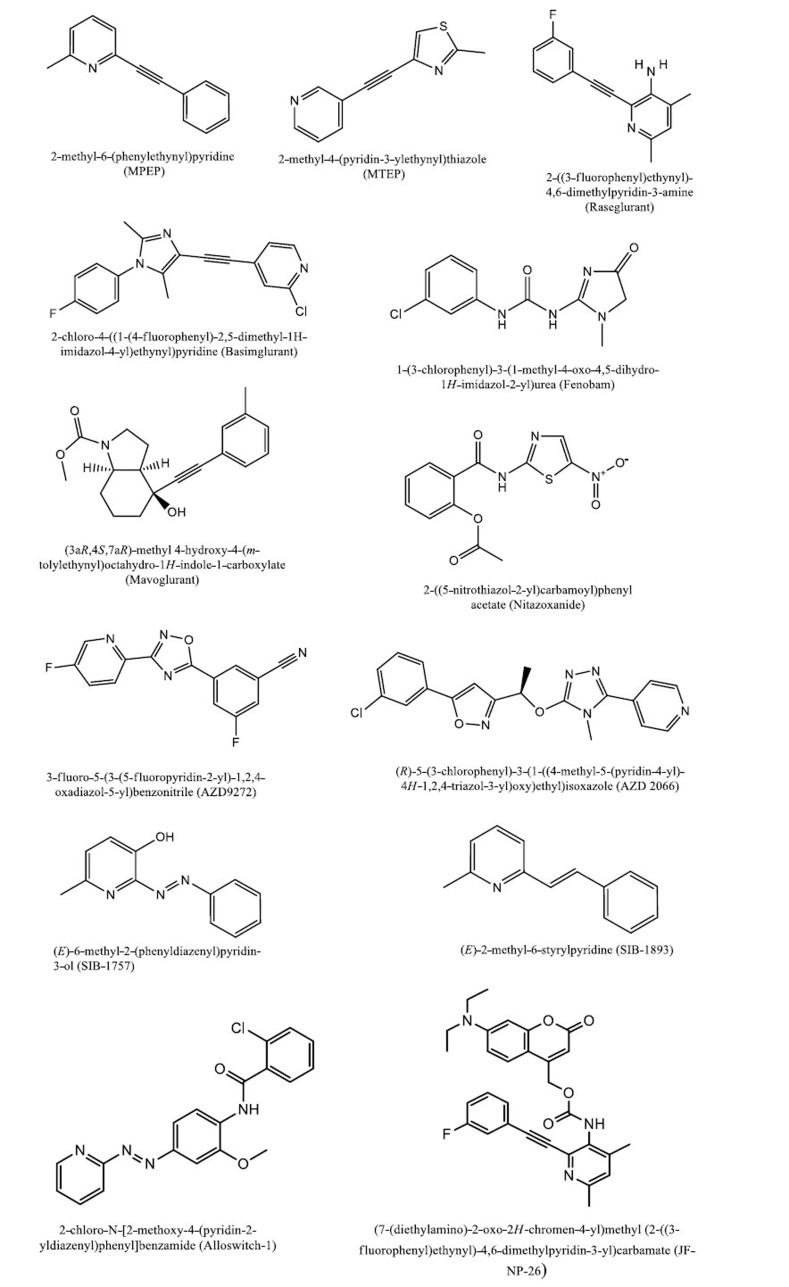
Some examples of mGlu5 NAMs chemical structures.

In response to the activation of mGlu5, multiple intracellular signaling pathways have the potential to modify neuronal excitability as well as synaptic plasticity. Activation of the mGlu5 leads to the production and maintenance of pain signals [145]. When the mGlu5 receptor is activated, multiple signaling pathways are triggered (Fig. **3**). These include the Phosphorylated-ERK1/2 pathway and Protein kinase C (PKC) pathway [114, 148]. Synaptic plasticity, gene expression, and the excitability of neurons are all altered as a result of these pathways, which in turn affects pain transmission and sensitization.

#### 3.1.1 Protein Kinase C (PKC) Pathway

mGlu5 receptors are activated when particular ligands, like glutamate or other agonists, bind to the extracellular domain of the receptor. When presynaptic terminals are stimulated, glutamate is released into the surrounding environment and binds to mGlu5 receptors. When mGlu5 is activated, it binds to Gαq/11 to trigger phospholipase Cβ1-related pathways, resulting in the production of inositol-1,4,5-triphosphate (IP3), diacylglycerol and protein kinase C (PKC) activation, among others [149]. IP3 triggers calcium-dependent ion channels and releases calcium from intracellular repositories. The intracellular calcium then activates protein kinase C (PKC) and its associated downstream signaling pathways, hence increasing PKC activity [150, 151]. A study conducted by Xie *et al*. (2017), investigated the role of the mGlu5 in the spinal dorsal horn in relation to paclitaxel-induced neuropathic pain [152]. The researchers discovered that in paclitaxel-induced neuropathic pain, presynaptic mGlu5 interacts with PKC and NMDARs to generate a signaling cascade that sustains a sustained increase of synaptic glutamate release to spinal dorsal horn neurons.

#### 3.1.2 Phosphorylated-ERK1/2 Pathway

Phosphorylation of ERK1/2 (extracellular signal-regulated kinase 1/2), which is a member of the mitogen-activated protein kinase (MAPK) family, has been associated with pain sensitization and the onset of neuropathic pain. In nerve injury-induced nociceptive hypersensitivity, nuclear mGlu5 expression is increased, and the phosphorylated-ERK1/2 pathway is activated [87]. The phosphorylation of ERK1/2 is a necessary step that must take place before the ERK pathway can be activated by mGlu5 receptors. Because of its function as a regulator of gene expression and neuronal plasticity, ERK1/2 phosphorylation is responsible for both the development of pain sensitization and the maintenance of chronic pain [153, 154].

#### 3.1.3 PI3K/Akt Pathway

When mGlu5 receptors are activated, NF-κB (nuclear factor kappa-light-chain-enhancer of activated B cells) can be activated *via* the Akt-NF-κB pathway. The transcription factor NF-κB is responsible for regulating the expression of genes that cause inflammation and pain sensitivity. It has been hypothesized that the activation of NF-κB in astrocytes is one of the factors that leads to the beginning of neuropathic pain. Evoked mGlu5 receptors in the PI3K/Akt pathway were shown to have neuroprotective effects [155]. Phosphoinositide 3-kinase (PI3K) is an upstream regulator of the protein kinase B (Akt) downstream effector, which plays a role in the regulation of cell survival and anti-apoptotic signaling. By activating Akt, one can see a reduction in both the neuroinflammatory response and pain sensitivity [156]. In addition, research has shown that there are interactions that take place between mGlu5 receptors and Homer proteins, which are a type of scaffold protein.

## 4 PHARMACOLOGICAL MODULATION OF MGLU5 ACTIVITY

The pharmacological modulation of mGlu5 activity holds promise for the diagnosis and treatment of a variety of neurological conditions. Because of the potential therapeutic utility of targeting this receptor's modulatory sites in the treatment of conditions as diverse as neurodegenerative illnesses and chronic pain, research into the effects of positive and negative allosteric modulators on mGlu5 is substantial (Table **3**). The mGlu5 function, achieved through the use of positive allosteric modulators (PAMs) or negative allosteric modulators (NAMs), has demonstrated potential in both preclinical and clinical investigations for the treatment of diverse disorders. The correction of hyperactivity associated with diseases such as neuropathic pain has been shown with the inhibition of mGlu5 [157-159]. Several studies have investigated the potential of pharmacological modulation using allosteric modulators of metabotropic glutamate 5 receptors in the context of pain modulation. This approach has been explored as a potential strategy for pain management.

### 4.1 Challenges of Targeting mGlu5 for Neuropathic Pain Treatment

Neuropathic pain is a complicated disorder characterized by various underlying causes. Targeting one specific receptor, such as mGlu5, for the treatment of neuropathic pain may lack specificity, which could result in undesired side effects or alteration of the receptor's normal function. The mGlu5 receptors have a broad distribution in the CNS, and their modification might result in diverse adverse effects connected to the CNS. Possible side effects of these drugs may include symptoms such as dizziness, sedation, decreased cognitive abilities, and alterations in emotional state [164, 165]. Developing mGlu5-targeted therapeutics presents a difficulty in balancing the desired analgesic effects with the possibility of associated side effects. Chronic pain syndromes, such as neuropathic pain, exhibit differences in receptor pharmacology and responsiveness to various therapies. The effectiveness of mGlu5 antagonist medication can vary among individuals, and not all patients may get substantial pain reduction. Designing pharmaceuticals that specifically target mGlu5 receptors while avoiding interference with other receptor subtypes or physiological processes is an obstacle to overcome. Developing a high level of selectivity is of utmost importance in order to prevent any possible off-target effects and undesired side effects. Although preclinical studies have demonstrated encouraging outcomes, the process of applying the effectiveness of mGlu5 regulation from animal models to humans has proven to be challenging in clinics [165]. Studies studying mGlu5 antagonists for neuropathic pain have not consistently shown substantial pain reduction or improvement in patient outcomes. Furthermore, certain mGlu5 antagonists have exhibited possible safety issues during therapeutic trials. For instance, the use of the mGlu5 antagonist raseglurant contributed to liver toxicity, which led to the termination of its clinical progression for the treatment of pain [129].

Repeatedly usage of mGlu5 modulators can result in a rise of tolerance [164], characterized by a decrease in the therapeutic effects over a period of time. Moreover, the sudden cessation of mGlu5 modulators might lead to withdrawal symptoms, suggesting a likelihood of dependence. Effectively managing tolerance and dependence poses a significant problem when using these medicines repeatedly and over an extended period of time. Accurate determination of the most effective dosage and administration strategy for mGlu5 modulators is essential in order to achieve the desired therapeutic effects. The challenge of achieving the optimal balance between providing effective pain relief while minimizing the occurrence of side effects is a challenge that must be addressed in clinical studies.

### 4.2 Negative Allosteric Modulators (NAMs) of mGlu5

Negative allosteric modulators (NAMs) are pharmacological compounds that have the ability to impede the activation of mGlu5. Additionally, they exhibit affinity for an alternative binding location on the receptor, resulting in a decrease in receptor function, while some NAMs have been shown to promote neuroprotection [66]. Several selective NAMs targeting mGlu5 have been discovered and examined. Some examples of these include SIB-1757, SIB-1893, 2-Methyl-6-(phenylethynyl)pyridine (MPEP), 3-((2-Methyl-4-thiazolyl)ethynyl)pyridine (MTEP) and Basimglurant [90]. These molecules have demonstrated enhanced effectiveness, selectivity, and ability to cross the blood-brain barrier. Furthermore, their potential therapeutic effects in treating inflammation and neuropathic pain have also been subject to investigation [166]. MPEP has the ability to bind to mGlu5 and negatively modulate its activity [167]. The compound is also an inverse agonist of mGlu5 and has been extensively studied for its possible therapeutic uses in several medical diseases. While one of the early mGlu5 NAMs to be used in the clinic was raseglurant (ADX10059), and another one was fenobam. Fenobam is a pharmacological compound utilized as an anxiolytic drug [168, 169]. Raseglurant and other selective NAMs of mGlu5 have repeatedly shown analgesic effects in experimental animal models of chronic pain [170]. These novel analgesic medications (NAMs) have demonstrated promise in the management of neuropathic pain and migraine. On the other hand, side effects associated with mechanisms may limit their systemic usage [129].

In addition, AZD9272 and AZD2066 are the other NAMs of the mGlu5. Both of them are antagonists of the mGlu5 receptor and have the ability to selectively bind to it and penetrate the central nervous system effectively [171]. These compounds have been studied for their possible application in pain models. A clinical trial examined the effectiveness of AZD2066 as a pain reliever for individuals suffering from severe diabetic neuropathy [172]. Research has indicated that both AZD9272 and AZD2066 demonstrate discriminative effects that are comparable to those of other mGlu5 antagonists [171].

Computational studies have shown that nitazoxanide has the ability to function as a NAM of the mGlu5 [173]. It was discovered in an *in silico* study that tizoxanide, the main active metabolite of nitazoxanide, fit *in silico* pharmacophore models constructed for both mGlu1 and mGlu5. Tizoxixanide had significant antagonist activity for both mGlu1 and mGlu5, as demonstrated by functional studies. The effectiveness of nitazoxanide given intraperitoneally in a rat model of neuropathic pain was reported [173]. However, further research is required to fully understand nitazoxanide's potential as mGlu5 NAM. The computational studies reveal intriguing insights into the potential pharmacological effects of nitazoxanide on the mGlu5 receptor. Nevertheless, it is crucial to acknowledge that computational studies are preliminary, and additional experimental studies are needed to fully verify these findings and study the clinical effects [173].

### 4.3 Novel Approaches to Treating Neuropathic Pain Targeting mGlu5

Prospective therapeutic approaches aimed at modulating the mGlu5 hold promise as potential approaches for the effective management of neuropathic pain. Although research in this area is still underway, preclinical studies have demonstrated encouraging outcomes with mGlu5 antagonists, positive allosteric modulators, and selective agonists. The utilization of mGlu5-targeting techniques in combination therapies may potentially enhance the efficacy of pain management.

#### 4.3.1 Combination Therapies

Combining mGlu5-targeting agents with other drugs or treatment modalities is another prospective strategy. Combination therapies have the potential to offer enhanced pain relief by selectively targeting various components implicated in neuropathic pain. One potential approach that has been investigated is the utilization of combination therapy, including mGlu5 antagonists and opioids. This strategy aims to enhance the analgesic properties of opioids while concurrently mitigating the adverse effects and potential for drug abuse [174-176]. This has the potential to yield greater pain alleviation in comparison to the exclusive targeting of mGlu5 alone. For instance, a research study designed the use of bivalent ligands, including a mu opioid agonist and mGlu5 antagonist, to target a MOR-mGlu5 heteromer for the treatment of pain. They administrated morphine, along with an mGlu5 antagonist (MPEP) [177]. This co-administration has been reported to have enhanced morphine antinociception with minimal or no adverse effects, such as morphine dependence. This suggested that both substances may possess analgesic characteristics and targeting the MOR-mGlu5 heteromer, could be a novel strategy for pain treatment. Current research is exploring the potential of combination therapies that involve targeting medications for mGlu5 together with other pain management strategies, such as nonsteroidal anti-inflammatory medicines (NSAIDs) or physical therapy.

## 5 DISCUSSION

The potential therapeutic application of modulating mGlu5 receptors shows promise in the management of neuropathic pain. Researchers are currently investigating both pharmacological drugs and non-pharmacological therapies as potential approaches to target mGlu5 and mitigate pain feelings. The efficacy and safety of these therapies are being further elucidated by ongoing clinical trials and research developments. Despite the presence of ongoing difficulties and unsolved inquiries, the utilization of mGlu5 regulation in the realm of neuropathic pain administration holds promise for those individuals who are in search of alleviation from this incapacitating ailment. By conducting further investigations into the complexities of mGlu5 signaling and its interactions with other pain regulation systems, we can lay the groundwork for the development of more efficient and specific therapeutic approaches in the future.

Neuropathic pain, a multifaceted and individualized sensation, presents a considerable obstacle to the healthcare sector. The search for better pain relief has prompted the investigation of new treatment options, with a focus on mGlu5. Combination therapies, including mGlu5 and other pain-modulating pathways, are under considerable investigation. Some studies have demonstrated beneficial combined effects, whereas others have shown complex and diverse results. A thorough comprehension of the interaction between mGlu5 and other pain-regulating systems is crucial due to the delicate equilibrium between treatment effectiveness and possible negative outcomes. Nevertheless, numerous challenges and considerations arise despite the tremendous promises. Clinical trials using NAMs targeting mGlu5 have faced challenges related to target involvement, treatment response evaluation, and the emergence of treatment resistance with prolonged drug use. Furthermore, the practicality of using medications that target mGlu5 is hindered by the possibility of adverse effects such as cognitive impairment and psychotomimetic symptoms. The problems highlight the critical importance of careful planning and strategic navigating in the development and clinical use of mGlu5-targeting combination treatments. The advancement of pain management includes researching combination therapies that target mGlu5, proving a dedication to reducing human suffering and improving quality of life.

### 5.1 Advantages and Limitations of Targeting mGlu5

Targeting the mGlu5 presents novel opportunities for addressing neuropathic pain. By directly targeting this receptor, drugs have the potential to offer more precise and efficient pain treatment. Furthermore, mGlu5 modulators may have a reduced propensity for addiction and dependency in contrast to conventional analgesic drugs. Moreover, through their impact on the fundamental mechanisms of neuropathic pain, drugs centered on mGlu5 have the capacity to offer enduring alleviation and enhance the quality of life for persons enduring chronic pain. Nevertheless, as with any other aspect of existence, there exist certain constraints. Due to the intricate structure of pain pathways and variations in individuals' responses to drugs, it is possible that not all individuals will experience benefits from targeting mGlu5. Current research endeavors to tackle these difficulties and enhance the application of mGlu5 modulators for pain control.

## CONCLUSION

In conclusion, although the targeting of mGlu5 shows potential for the treatment of neuropathic pain, additional study is required to comprehensively comprehend its prospective advantages and constraints. Further research is necessary to determine the long-term safety and effectiveness of this therapy, as well as to improve treatment methods. Given the continuous progress and forthcoming studies in the field, it is highly likely that mGlu5 could be the crucial factor in developing more efficient and specific approaches for managing neuropathic pain in the future.

## Figures and Tables

**Table 1:** The table shows some of the alterations at various sites that contribute to the generation of neuropathic pain.

**Model**	**Disease**	**Site**	**Alterations**	**References**
Rats Rodents Humans	Neuropathic pain Neuropathic pain Reflex sympathetic dystrophy (RSD)	Peripheral Fibers	Increased nociceptor sensitivity Ectopic firing Altered signal transmission	[11] [12] [21]
Rodents	Neuropathic pain	Dorsal Root Ganglion	Increased excitability Altered gene expression Ectopic firing	[13]
Mice Humans-	Neuropathic pain Postherpetic neuralgia	Spinal Cord	Inflammation mediator release Glial cell activation Increased synaptic efficacy Decreased inhibitory tone Dorsal horn remodelling	[22] [19] [20, 23]
Humans	Fibromyalgia and Chronic Fatigue Syndrome	Brain	Inflammation mediator release Glial cell activation Cortical remodelling Increased descending facilitation Decreased descending inhibition	[18] [17, 24]

**Table 2:** Effective doses of different drugs and their adverse effects in neuropathic pain management. (Mg/d= milligram per day).

**Drug**	**Effective Dose**	**Adverse Effects**	**References**
Gabapentin	900-3,600 mg/d in divided doses	Sedation, dizziness, weight gain and peripheral edema	[36, 42, 57, 58]
Pregabalin	150-600 mg/d in divided doses		
Amitriptyline	50-150 mg/d	Anticholinergic effect and weight gain	[57]
Nortriptyline	75-100 mg/d	Nausea, constipation, abdominal pain and hypertension at high doses of venlafaxine	[36, 59]
Duloxetine	60-120 mg/d		
Venlafaxine	75-225 mg/d		[60]
Carbamazepine	400-800 mg/d in divided doses	Impaired mental and motor function	[33]
Tramadol	50-400 mg/d	Nausea, constipation, dizziness and vomiting	[33, 36]
Topical agents (5% lidocaine patches & 8% capsaicin)	Apply lidocaine patches for an hour and capsaicin cream for 12 hours	Erythema, itching and pain with no rash	[36]

**Table 3:** Pain modulation in different animal models by mGlu5 ligands.

**-**	**Drugs**	**Localization**	**Animal Model**	**Effects**	**References**
Orthosteric agonist	DHPG S-DHPG	Spinal cord PAG	Naïve Rats Naïve Mice	Decreased the hind limb withdrawal threshold Decreased thermal hyperalgesia	[153] [119]
Allosteric modulators	Alloswitch-1	Amygdala	Mice	Restore mechanical sensitivity	[160]
	S-4CPG Fenobam MPEP	Spinal cord	CCI Rats SNI Rats CFA	Attenuation of mechanical allodynia and cold hyperalgesia Reduction of glutamate-induced spontaneous pain behavior and mechanical allodynia Reversed pain hypersensitivity	[161] [87] [162]
	MPEP	-	Carrageenan rats CFA	Reversal of inflammatory hyperalgesia Decrease thermal hyperalgesia	[147] [112]
	MPEP	-	CFA Rats	Reversal of mechanical hyperalgesia	[147]
	Fenobam	-	CFA Mice	Reduce thermal hypersensitivity	[163]
	MPEP	-	CFA model carrageenan model	Decreased dose-dependent reversal of mechanical allodynia	[112]
	MPEP	Prefrontal cortex	SNL Rats	Decreased tactile hypersensitivity	[103]

## LIST OF ABBREVIATIONS

ATP: Adenosine Triphosphate
CCI: Chronic Constriction Injury
CNS: Central Nervous System
CS: Central Sensitization
DPN: Diabetic Polyneuropathy
DRG: Dorsal Root Ganglion
mGlu5: Metabotropic Glutamate Receptor 5
NAMs: Negative Allosteric Modulators
NNTs: Number-needed-to-treat
NP: Neuropathic Pain
NSAIDs: Nonsteroidal Anti-inflammatory Medicines
PAMs: Positive Allosteric Modulators
PHN: Post-herpetic Neuralgia
PI3K: Phosphoinositide 3-kinase
PKC: Protein Kinase C
SNRIs: Serotonin-norepinephrine Reuptake Inhibitors
TCAs: Tricyclic Antidepressants
VFT: Venus Flytrap Domain

## References

[1] Bouhassira D. (2019). Neuropathic pain: Definition, assessment and epidemiology.. Rev. Neurol..

[2] Colloca L., Ludman T., Bouhassira D., Baron R., Dickenson A.H., Yarnitsky D., Freeman R., Truini A., Attal N., Finnerup N.B., Eccleston C., Kalso E., Bennett D.L., Dworkin R.H., Raja S.N. (2017). Neuropathic pain.. Nat. Rev. Dis. Primers.

[3] Dupoiron D., Brill S., Eeltink C., Barragán B., Bell D., Petersen G., Eerdekens M., Ryan D., Rakuša M. (2022). Diagnosis, management and impact on patients’ lives of cancer‐related neuropathic pain (CRNP): A European survey.. Eur. J. Cancer Care (Engl.).

[4] Irving G.A. (2005). Contemporary assessment and management of neuropathic pain.. Neurology.

[5] Aley K.O., Reichling D.B., Levine J.D. (1996). Vincristine hyperalgesia in the rat: A model of painful vincristine neuropathy in humans.. Neuroscience.

[6] Finnerup N.B., Kuner R., Jensen T.S. (2021). Neuropathic pain: From mechanisms to treatment.. Physiol. Rev..

[7] He F., Gu X-S., Chu X-L., Song X-Z., Li Q., Li Y-R., Ming D. (2022). Basic mechanisms of peripheral nerve injury and treatment *via* electrical stimulation.. Neural Regen. Res..

[8] Boyd A., Byrne S., Middleton R.J., Banati R.B., Liu G.J. (2021). Control of neuroinflammation through radiation-induced microglial changes.. Cells.

[9] Inoue K., Tsuda M. (2018). Microglia in neuropathic pain: Cellular and molecular mechanisms and therapeutic potential.. Nat. Rev. Neurosci..

[10] Navarro X. (2009). Neural plasticity after nerve injury and regeneration.. Int. Rev. Neurobiol..

[11] Chaban VV (2010). Peripheral sensitization of sensory neurons Ethn Dis.

[12] Meacham K., Shepherd A., Mohapatra D.P., Haroutounian S. (2017). Neuropathic pain: Central vs. peripheral mechanisms.. Curr. Pain Headache Rep..

[13] Sharma A., Behl T., Sharma L., Shah O.P., Yadav S., Sachdeva M., Rashid S., Bungau S.G., Bustea C. (2023). Exploring the molecular pathways and therapeutic implications of angiogenesis in neuropathic pain.. Biomed. Pharmacother..

[14] Novakovic S.D., Tzoumaka E., McGivern J.G., Haraguchi M., Sangameswaran L., Gogas K.R., Eglen R.M., Hunter J.C. (1998). Distribution of the tetrodotoxin-resistant sodium channel PN3 in rat sensory neurons in normal and neuropathic conditions.. J. Neurosci..

[15] Latremoliere A., Woolf C.J. (2009). Central sensitization: A generator of pain hypersensitivity by central neural plasticity.. J. Pain.

[16] Nijs J., Van Houdenhove B., Oostendorp R.A.B. (2010). Recognition of central sensitization in patients with musculoskeletal pain: Application of pain neurophysiology in manual therapy practice.. Man. Ther..

[17] Meeus M., Nijs J., Van de Wauwer N., Toeback L., Truijen S. (2008). Diffuse noxious inhibitory control is delayed in chronic fatigue syndrome: An experimental study.. Pain.

[18] Meeus M., Nijs J. (2007). Central sensitization: A biopsychosocial explanation for chronic widespread pain in patients with fibromyalgia and chronic fatigue syndrome.. Clin. Rheumatol..

[19] Fields H.L., Rowbotham M., Baron R. (1998). Postherpetic neuralgia: Irritable nociceptors and deafferentation.. Neurobiol. Dis..

[20] Woolf C.J., Shortland P., Coggeshall R.E. (1992). Peripheral nerve injury triggers central sprouting of myelinated afferents.. Nature.

[21] Gracely R.H., Lynch S.A., Bennett G.J. (1992). Painful neuropathy: Altered central processing maintained dynamically by peripheral input.. Pain.

[22] Ohsawa M., Yamamoto S., Ono H. (2014). Contribution of the sensitization of supraspinal nociceptive transmission in chronic pain.. Yakugaku Zasshi.

[23] West S.J., Bannister K., Dickenson A.H., Bennett D.L. (2015). Circuitry and plasticity of the dorsal horn – Toward a better understanding of neuropathic pain.. Neuroscience.

[24] Millan M.J. (2002). Descending control of pain.. Prog. Neurobiol..

[25] Dworkin R.H., O’Connor A.B., Audette J., Baron R., Gourlay G.K., Haanpää M.L., Kent J.L., Krane E.J., LeBel A.A., Levy R.M., Mackey S.C., Mayer J., Miaskowski C., Raja S.N., Rice A.S.C., Schmader K.E., Stacey B., Stanos S., Treede R.D., Turk D.C., Walco G.A., Wells C.D. (2010). Recommendations for the pharmacological management of neuropathic pain: An overview and literature update.. Mayo Clin. Proc..

[26] Fornasari D. (2017). Pharmacotherapy for neuropathic pain: A review.. Pain Ther..

[27] (2013). Neuropathic pain in adults: Pharmacological management in nonspecialist settings. NICE, Clinical Guideline.. http://nice.org.uk/guidance/cg173.

[28] Finnerup N.B., Attal N., Haroutounian S., McNicol E., Baron R., Dworkin R.H., Gilron I., Haanpää M., Hansson P., Jensen T.S., Kamerman P.R., Lund K., Moore A., Raja S.N., Rice A.S.C., Rowbotham M., Sena E., Siddall P., Smith B.H., Wallace M. (2015). Pharmacotherapy for neuropathic pain in adults: A systematic review and meta-analysis.. Lancet Neurol..

[29] Sumitani M., Sakai T., Matsuda Y., Abe H., Yamaguchi S., Hosokawa T., Fukui S. (2018). Executive summary of the clinical guidelines of pharmacotherapy for neuropathic pain: Second edition by the Japanese society of pain clinicians.. J. Anesth..

[30] Mu A., Weinberg E., Moulin D.E., Clarke H. (2017). Pharmacologic management of chronic neuropathic pain: Review of the Canadian Pain Society consensus statement.. Can. Fam. Physician.

[31] Attal N., Cruccu G., Baron R., Haanpää M., Hansson P., Jensen T.S., Nurmikko T. (2010). EFNS guidelines on the pharmacological treatment of neuropathic pain: 2010 revision.. Eur. J. Neurol..

[32] Guidelines for the pharmacological treatment of neuropathic pain Australian Clinical Practice Guidelines.. https://www.clinicalguidelines.gov.au/portal/2290/guidelines-treatment-neuropathic-pain.

[33] Thouaye M., Yalcin I. (2023). Neuropathic pain: From actual pharmacological treatments to new therapeutic horizons.. Pharmacol. Ther..

[34] Horishita T., Yanagihara N., Ueno S., Okura D., Horishita R., Minami T., Ogata Y., Sudo Y., Uezono Y., Sata T., Kawasaki T. (2017). Antidepressants inhibit Nav1.3, Nav1.7, and Nav1.8 neuronal voltage-gated sodium channels more potently than Nav1.2 and Nav1.6 channels expressed in Xenopus oocytes.. Naunyn Schmiedebergs Arch. Pharmacol..

[35] Obata H. (2017). Analgesic mechanisms of antidepressants for neuropathic pain.. Int. J. Mol. Sci..

[36] Jensen T.S., Madsen C.S., Finnerup N.B. (2009). Pharmacology and treatment of neuropathic pains.. Curr. Opin. Neurol..

[37] Smith T., Nicholson R.A. (2007). Review of duloxetine in the management of diabetic peripheral neuropathic pain.. Vasc. Health Risk Manag..

[38] Moulin D.E., Boulanger A., Clark A.J., Clarke H., Dao T., Finley G.A., Furlan A., Gilron I., Gordon A., Morley-Forster P.K., Sessle B.J., Squire P., Stinson J., Taenzer P., Velly A., Ware M.A., Weinberg E.L., Williamson O.D. (2014). Pharmacological management of chronic neuropathic pain: Revised consensus statement from the Canadian Pain Society.. Pain Res. Manag..

[39] Li C.T., Watson J.C. (2019). Anticonvulsants in the Treatment of Pain..

[40] Jensen M.P., Chiang Y.K., Wu J. (2009). Assessment of pain quality in a clinical trial of gabapentin extended release for postherpetic neuralgia.. Clin. J. Pain.

[41] Irving G., Jensen M., Cramer M., Wu J., Chiang Y.K., Tark M., Wallace M. (2009). Efficacy and tolerability of gastric-retentive gabapentin for the treatment of postherpetic neuralgia: Results of a double-blind, randomized, placebo-controlled clinical trial.. Clin. J. Pain.

[42] Arezzo J.C., Rosenstock J., LaMoreaux L., Pauer L. (2008). Efficacy and safety of pregabalin 600 mg/d for treating painful diabetic peripheral neuropathy: A double-blind placebo-controlled trial.. BMC Neurol..

[43] Wiffen P.J., Derry S., Bell R.F., Rice A.S.C., Tölle T.R., Phillips T., Moore R.A. (2017). Gabapentin for chronic neuropathic pain in adults.. Cochrane Libr..

[44] Birse F., Derry S., Moore R.A. (2012). Phenytoin for neuropathic pain and fibromyalgia in adults.. Cochrane Libr..

[45] Wiffen P.J., Derry S., Moore R.A., Aldington D., Cole P., Rice A.S.C., Lunn M.P.T., Hamunen K., Haanpaa M., Kalso E.A. (2013). Antiepileptic drugs for neuropathic pain and fibromyalgia - An overview of Cochrane reviews.. Cochrane Libr..

[46] (2022). In: Anesthesiology In-Training Exam Review..

[47] Wiffen P.J., Derry S., Moore R.A., Kalso E.A. (2014). Carbamazepine for chronic neuropathic pain and fibromyalgia in adults.. Cochrane Libr..

[48] Bates D., Schultheis B.C., Hanes M.C., Jolly S.M., Chakravarthy K.V., Deer T.R., Levy R.M., Hunter C.W. (2019). A comprehensive algorithm for management of neuropathic pain.. Pain Med..

[49] Hermanns H., Hollmann M.W., Stevens M.F., Lirk P., Brandenburger T., Piegeler T., Werdehausen R. (2019). Molecular mechanisms of action of systemic lidocaine in acute and chronic pain: A narrative review.. Br. J. Anaesth..

[50] Miclescu A., Schmelz M., Gordh T. (2015). Differential analgesic effects of subanesthetic concentrations of lidocaine on spontaneous and evoked pain in human painful neuroma: A randomized, double blind study.. Scand. J. Pain.

[51] Sommer C., Cruccu G. (2017). Topical treatment of peripheral neuropathic pain: Applying the evidence.. J. Pain Symptom Manage..

[52] Derry S., Wiffen P.J., Kalso E.A., Bell R.F., Aldington D., Phillips T., Gaskell H., Moore R.A. (2017). Topical analgesics for acute and chronic pain in adults - An overview of cochrane reviews.. Cochrane Libr..

[53] Anand P., Privitera R., Donatien P., Fadavi H., Tesfaye S., Bravis V., Misra V.P. (2022). Reversing painful and non-painful diabetic neuropathy with the capsaicin 8% patch: Clinical evidence for pain relief and restoration of function *via* nerve fiber regeneration.. Front. Neurol..

[54] Dludla P.V., Nkambule B.B., Cirilli I., Marcheggiani F., Mabhida S.E., Ziqubu K., Ntamo Y., Jack B., Nyambuya T.M., Hanser S., Mazibuko-Mbeje S.E. (2022). Capsaicin, its clinical significance in patients with painful diabetic neuropathy.. Biomed. Pharmacother..

[55] Casale R., Symeonidou Z., Bartolo M. (2017). Topical treatments for localized neuropathic pain.. Curr. Pain Headache Rep..

[56] Lourenco J.L., Campelo Feres C., Telles-Dias P.R. (2010). Topical preparations for pain relief: Efficacy and patient adherence.. J. Pain Res..

[57] Gilron I., Bailey J.M., Tu D., Holden R.R., Jackson A.C., Houlden R.L. (2009). Nortriptyline and gabapentin, alone and in combination for neuropathic pain: A double-blind, randomised controlled crossover trial.. Lancet.

[58] Siddall P.J., Cousins M.J., Otte A., Griesing T., Chambers R., Murphy T.K. (2006). Pregabalin in central neuropathic pain associated with spinal cord injury.. Neurology.

[59] Sindrup S.H., Otto M., Finnerup N.B., Jensen T.S. (2005). Antidepressants in the treatment of neuropathic pain.. Basic Clin. Pharmacol. Toxicol..

[60] Rowbotham M.C., Goli V., Kunz N.R., Lei D. (2004). Venlafaxine extended release in the treatment of painful diabetic neuropathy: A double-blind, placebo-controlled study.. Pain.

[61] Sindrup S.H., Andersen G., Madsen C., Smith T., Brøsen K., Jensen T.S. (1999). Tramadol relieves pain and allodynia in polyneuropathy: A randomised, double-blind, controlled trial.. Pain.

[62] Boureau F., Legallicier P., Kabir-Ahmadi M. (2003). Tramadol in post-herpetic neuralgia: A randomized, double-blind, placebo-controlled trial.. Pain.

[63] Arbaiza D., Vidal O. (2007). Tramadol in the treatment of neuropathic cancer pain: A double-blind, placebo-controlled study.. Clin. Drug Investig..

[64] Freo U., Romualdi P., Kress H.G. (2019). Tapentadol for neuropathic pain: A review of clinical studies.. J. Pain Res..

[65] Schwartz S., Etropolski M., Shapiro D.Y., Okamoto A., Lange R., Haeussler J., Rauschkolb C. (2011). Safety and efficacy of tapentadol ER in patients with painful diabetic peripheral neuropathy: Results of a randomized-withdrawal, placebo-controlled trial.. Curr. Med. Res. Opin..

[66] Azam S., Jakaria M., Kim J., Ahn J., Kim I.S., Choi D.K. (2022). Group I mGluRs in therapy and diagnosis of Parkinson’s disease: Focus on mGluR5 subtype.. Biomedicines.

[67] Collingridge G.L., Olsen R.W., Peters J., Spedding M. (2009). A nomenclature for ligand-gated ion channels.. Neuropharmacology.

[68] Doré A.S., Okrasa K., Patel J.C., Serrano-Vega M., Bennett K., Cooke R.M., Errey J.C., Jazayeri A., Khan S., Tehan B., Weir M., Wiggin G.R., Marshall F.H. (2014). Structure of class C GPCR metabotropic glutamate receptor 5 transmembrane domain.. Nature.

[69] Kolber B.J., Montana M.C., Carrasquillo Y., Xu J., Heinemann S.F., Muglia L.J., Gereau R.W. (2010). Activation of metabotropic glutamate receptor 5 in the amygdala modulates pain-like behavior.. J. Neurosci..

[70] Mazzitelli M., Palazzo E., Maione S., Neugebauer V., Group I.I. (2018). Group II metabotropic glutamate receptors: Role in pain mechanisms and pain modulation.. Front. Mol. Neurosci..

[71] Hallock R.M., Martyniuk C.J., Finger T.E. (2009). Group III metabotropic glutamate receptors (mGluRs) modulate transmission of gustatory inputs in the brain stem.. J. Neurophysiol..

[72] Grueter B.A., Winder D.G. (2009). In: Encyclopedia of Neuroscience..

[73] Wright R.A., Johnson B.G., Zhang C., Salhoff C., Kingston A.E., Calligaro D.O., Monn J.A., Schoepp D.D., Marek G.J. (2013). CNS distribution of metabotropic glutamate 2 and 3 receptors: Transgenic mice and [3H]LY459477 autoradiography.. Neuropharmacology.

[74] Gu G., Lorrain D.S., Wei H., Cole R.L., Zhang X., Daggett L.P., Schaffhauser H.J., Bristow L.J., Lechner S.M. (2008). Distribution of metabotropic glutamate 2 and 3 receptors in the rat forebrain: Implication in emotional responses and central disinhibition.. Brain Res..

[75] Srivastava A., Das B., Yao A.Y., Yan R. (2020). Metabotropic glutamate receptors in Alzheimer’s disease synaptic dysfunction: Therapeutic opportunities and hope for the future.. J. Alzheimers Dis..

[76] Mao L.M., Mathur N., Mahmood T., Rajan S., Chu X.P., Wang J.Q. (2022). Phosphorylation and regulation of group II metabotropic glutamate receptors (mGlu2/3) in neurons.. Front. Cell Dev. Biol..

[77] Neugebauer V. (2008). In: The Glutamate Receptors..

[78] Malherbe P., Kew J.N.C., Richards J.G., Knoflach F., Kratzeisen C., Zenner M.T., Faull R.L.M., Kemp J.A., Mutel V. (2002). Identification and characterization of a novel splice variant of the metabotropic glutamate receptor 5 gene in human hippocampus and cerebellum.. Brain Res. Mol. Brain Res..

[79] Minakami R., Iida K., Hirakawa N., Sugiyama H. (1995). The expression of two splice variants of metabotropic glutamate receptor subtype 5 in the rat brain and neuronal cells during development.. J. Neurochem..

[80] Joly C., Gomeza J., Brabet I., Curry K., Bockaert J., Pin J.P. (1995). Molecular, functional, and pharmacological characterization of the metabotropic glutamate receptor type 5 splice variants: Comparison with mGluR1.. J. Neurosci..

[81] Alvarez F.J., Villalba R.M., Carr P.A., Grandes P., Somohano P.M. (2000). Differential distribution of metabotropic glutamate receptors 1a, 1b, and 5 in the rat spinal cord.. J. Comp. Neurol..

[82] Shigemoto R., Nomura S., Ohishi H., Sugihara H., Nakanishi S., Mizuno N. (1993). Immunohistochemical localization of a metabotropic glutamate receptor, mGluR5, in the rat brain.. Neurosci. Lett..

[83] Romano C., Sesma M.A., McDonald C.T., O’malley K., van den Pol A.N., Olney J.W. (1995). Distribution of metabotropic glutamate receptor mGluR5 immunoreactivity in rat brain.. J. Comp. Neurol..

[84] Hoffpauir B.K., Gleason E.L. (2002). Activation of mGluR5 modulates GABA(A) receptor function in retinal amacrine cells.. J. Neurophysiol..

[85] Schoepp D.D. (2001). Unveiling the functions of presynaptic metabotropic glutamate receptors in the central nervous system.. J. Pharmacol. Exp. Ther..

[86] Budgett R.F., Bakker G., Sergeev E., Bennett K.A., Bradley S.J. (2022). Targeting the type 5 metabotropic glutamate receptor: A potential therapeutic strategy for neurodegenerative diseases?. Front. Pharmacol..

[87] Vincent K., Cornea V.M., Jong Y.J.I., Laferrière A., Kumar N., Mickeviciute A., Fung J.S.T., Bandegi P., Ribeiro-da-Silva A., O’Malley K.L., Coderre T.J. (2016). Intracellular mGluR5 plays a critical role in neuropathic pain.. Nat. Commun..

[88] Wu H., Wang C., Gregory K.J., Han G.W., Cho H.P., Xia Y., Niswender C.M., Katritch V., Meiler J., Cherezov V., Conn P.J., Stevens R.C. (2014). Structure of a class C GPCR metabotropic glutamate receptor 1 bound to an allosteric modulator.. Science.

[89] Ohashi H., Maruyama T., Higashi-Matsumoto H., Nomoto T., Takeuchi Y., Takeuchi Y. (2002). A novel binding assay for metabotropic glutamate receptors using [3H] L-quisqualic acid and recombinant receptors.. Z. Naturforsch. C J. Biosci..

[90] Niswender C.M., Conn P.J. (2010). Metabotropic glutamate receptors: Physiology, pharmacology, and disease.. Annu. Rev. Pharmacol. Toxicol..

[91] Huang S., Cao J., Jiang M., Labesse G., Liu J., Pin J.P., Rondard P. (2011). Interdomain movements in metabotropic glutamate receptor activation.. Proc. Natl. Acad. Sci. USA.

[92] Seven A.B., Barros-Álvarez X., de Lapeyrière M., Papasergi-Scott M.M., Robertson M.J., Zhang C., Nwokonko R.M., Gao Y., Meyerowitz J.G., Rocher J.P., Schelshorn D., Kobilka B.K., Mathiesen J.M., Skiniotis G. (2021). G-protein activation by a metabotropic glutamate receptor.. Nature.

[93] Nasrallah C., Cannone G., Briot J., Rottier K., Berizzi A.E., Huang C.Y., Quast R.B., Hoh F., Banères J.L., Malhaire F., Berto L., Dumazer A., Font-Ingles J., Gómez-Santacana X., Catena J., Kniazeff J., Goudet C., Llebaria A., Pin J.P., Vinothkumar K.R., Lebon G. (2021). Agonists and allosteric modulators promote signaling from different metabotropic glutamate receptor 5 conformations.. Cell Rep..

[94] Koehl A., Hu H., Feng D., Sun B., Zhang Y., Robertson M.J., Chu M., Kobilka T.S., Laeremans T., Steyaert J., Tarrasch J., Dutta S., Fonseca R., Weis W.I., Mathiesen J.M., Skiniotis G., Kobilka B.K. (2019). Structural insights into the activation of metabotropic glutamate receptors.. Nature.

[95] Van Drie J.H., Tong L. (2020). Cryo-EM as a powerful tool for drug discovery.. Bioorg. Med. Chem. Lett..

[96] Shen S., Zhao C., Wu C., Sun S., Li Z., Yan W., Shao Z. (2023). Allosteric modulation of G protein-coupled receptor signaling.. Front. Endocrinol..

[97] Chen C.J., Jiang C., Yuan J., Chen M., Cuyler J., Xie X.Q., Feng Z. (2022). How do modulators affect the orthosteric and allosteric binding pockets?. ACS Chem. Neurosci..

[98] Jeffrey Conn P., Christopoulos A., Lindsley C.W. (2009). Allosteric modulators of GPCRs: A novel approach for the treatment of CNS disorders.. Nat. Rev. Drug Discov..

[99] Shipe W.D., Wolkenberg S.E., Williams D.L., Lindsley C.W. (2005). Recent advances in positive allosteric modulators of metabotropic glutamate receptors.. Curr. Opin. Drug Discov. Devel..

[100] Stansley B.J., Conn P.J. (2019). Neuropharmacological insight from allosteric modulation of mglu receptors.. Trends Pharmacol. Sci..

[101] Kim S.K., Hayashi H., Ishikawa T., Shibata K., Shigetomi E., Shinozaki Y., Inada H., Roh S.E., Kim S.J., Lee G., Bae H., Moorhouse A.J., Mikoshiba K., Fukazawa Y., Koizumi S., Nabekura J. (2016). Cortical astrocytes rewire somatosensory cortical circuits for peripheral neuropathic pain.. J. Clin. Invest..

[102] Bushnell M.C., Duncan G.H., Hofbauer R.K., Ha B., Chen J.I., Carrier B. (1999). Pain perception: Is there a role for primary somatosensory cortex?. Proc. Natl. Acad. Sci. USA.

[103] Danjo Y., Shigetomi E., Hirayama Y.J., Kobayashi K., Ishikawa T., Fukazawa Y., Shibata K., Takanashi K., Parajuli B., Shinozaki Y., Kim S.K., Nabekura J., Koizumi S. (2022). Transient astrocytic mGluR5 expression drives synaptic plasticity and subsequent chronic pain in mice.. J. Exp. Med..

[104] Fellin T., D’Ascenzo M., Haydon P.G. (2007). Astrocytes control neuronal excitability in the nucleus accumbens.. ScientificWorldJournal.

[105] Mah W., Lee S.M., Lee J., Bae J.Y., Ju J.S., Lee C.J., Ahn D.K., Bae Y.C. (2017). A role for the purinergic receptor P2X3 in astrocytes in the mechanism of craniofacial neuropathic pain.. Sci. Rep..

[106] Chen C.C., Akopian A.N., Sivilottit L., Colquhoun D., Burnstock G., Wood J.N.A. (1995). P2X purinoceptor expressed by a subset of sensory neurons.. Nature.

[107] He Y.Q., Lang X.Q., Lin L., Ji L., Yuan X.Y., Chen Q., Ran Y.M., Chen H.S., Li L., Wang J.M., Wang Z.G., Gregersen H., Zou D.W., Liang H.P., Yang M. (2017). P2X3 receptor‐mediated visceral hyperalgesia and neuronal sensitization following exposure to PTSD ‐like stress in the dorsal root ganglia of rats.. Neurogastroenterol. Motil..

[108] Hu S., Sun Q., Du W.J., Song J., Li X., Zhang P.A., Xu J.T., Xu G.Y. (2020). Adult stress promotes purinergic signaling to induce visceral pain in rats with neonatal maternal deprivation.. Neurosci. Bull..

[109] Peavy R.D., Chang M.S.S., Sanders-Bush E., Conn P.J. (2001). Metabotropic glutamate receptor 5-induced phosphorylation of extracellular signal-regulated kinase in astrocytes depends on transactivation of the epidermal growth factor receptor.. J. Neurosci..

[110] Peavy R.D., Conn P.J. (1998). Phosphorylation of mitogen-activated protein kinase in cultured rat cortical glia by stimulation of metabotropic glutamate receptors.. J. Neurochem..

[111] Yu J., Zhao C., Luo X. (2013). The effects of electroacupuncture on the extracellular signal-regulated kinase 1/2/P2X3 signal pathway in the spinal cord of rats with chronic constriction injury.. Anesth. Analg..

[112] Walker K., Reeve A., Bowes M., Winter J., Wotherspoon G., Davis A., Schmid P., Gasparini F., Kuhn R., Urban L. (2001). mGlu5 receptors and nociceptive function II. mGlu5 receptors functionally expressed on peripheral sensory neurones mediate inflammatory hyperalgesia.. Neuropharmacology.

[113] Hama A. (2003). Acute activation of the spinal cord metabotropic glutamate subtype-5 receptor leads to cold hypersensitivity in the rat.. Neuropharmacology.

[114] Karim F., Wang C.C., Gereau R.W. (2001). IV Metabotropic glutamate receptor subtypes 1 and 5 are activators of extracellular signal-regulated kinase signaling required for inflammatory pain in mice.. J. Neurosci..

[115] Bhave G., Karim F., Carlton S.M., Gereau R.W. (2001). IV Peripheral group I metabotropic glutamate receptors modulate nociception in mice.. Nat. Neurosci..

[116] Palazzo E., Genovese R., Mariani L., Siniscalco D., Marabese I., de Novellis V., Rossi F., Maione S. (2004). Metabotropic glutamate receptor 5 and dorsal raphe serotonin release in inflammatory pain in rat.. Eur. J. Pharmacol..

[117] Palazzo E., Luongo L., Bellini G., Guida F., Marabese I., Boccella S., Rossi F., Maione S., de Novellis V. (2012). Changes in cannabinoid receptor subtype 1 activity and interaction with metabotropic glutamate subtype 5 receptors in the periaqueductal gray-rostral ventromedial medulla pathway in a rodent neuropathic pain model.. CNS Neurol. Disord. Drug Targets.

[118] Almeida-Santos A.F., Moreira F.A., Guimaraes F.S., Aguiar D.C. (2017). 2-Arachidonoylglycerol endocannabinoid signaling coupled to metabotropic glutamate receptor type-5 modulates anxiety-like behavior in the rat ventromedial prefrontal cortex.. J. Psychopharmacol..

[119] Maione S., Marabese I., Leyva J., Palazzo E., de Novellis V., Rossi F. (1998). Characterisation of mGluRs which modulate nociception in the PAG of the mouse.. Neuropharmacology.

[120] Chung G., Shim H.G., Kim C.Y., Ryu H.H., Jang D.C., Kim S.H., Lee J., Kim C.E., Kim Y.K., Lee Y.S., Kim J., Kim S.K., Worley P.F., Kim S.J. (2020). Persistent activity of metabotropic glutamate receptor 5 in the periaqueductal gray constrains emergence of chronic neuropathic pain.. Curr. Biol..

[121] Salt T.E., Binns K.E. (2000). Contributions of mGlu1 and mGlu5 receptors to interactions with N-methyl-d-aspartate receptor-mediated responses and nociceptive sensory responses of rat thalamic neurons.. Neuroscience.

[122] Carrasquillo Y., Gereau R.W. (2008). Hemispheric lateralization of a molecular signal for pain modulation in the amygdala.. Mol. Pain,.

[123] Veinante P., Yalcin I., Barrot M. (2013). The amygdala between sensation and affect: A role in pain.. J. Mol. Psychiatry.

[124] Li W., Neugebauer V. (2004). Differential roles of mGluR1 and mGluR5 in brief and prolonged nociceptive processing in central amygdala neurons.. J. Neurophysiol..

[125] Crock L.W., Kolber B.J., Morgan C.D., Sadler K.E., Vogt S.K., Bruchas M.R., Gereau R.W. (2012). IV Central amygdala metabotropic glutamate receptor 5 in the modulation of visceral pain.. J. Neurosci..

[126] Ji R.R. (2004). Peripheral and central mechanisms of inflammatory pain, with emphasis on MAP kinases.. Curr. Drug Targets Inflamm. Allergy.

[127] Mazzitelli M., Presto P., Antenucci N., Meltan S., Neugebauer V. (2022). Recent advances in the modulation of pain by the metabotropic glutamate receptors.. Cells.

[128] Nicoletti F., Bruno V., Ngomba R.T., Gradini R., Battaglia G. (2015). Metabotropic glutamate receptors as drug targets: what’s new?. Curr. Opin. Pharmacol..

[129] Font J., López-Cano M., Notartomaso S., Scarselli P., Di Pietro P., Bresolí-Obach R., Battaglia G., Malhaire F., Rovira X., Catena J., Giraldo J., Pin J.P., Fernández-Dueñas V., Goudet C., Nonell S., Nicoletti F., Llebaria A., Ciruela F. (2017). Optical control of pain *in vivo* with a photoactive mGlu5 receptor negative allosteric modulator.. eLife.

[130] Notartomaso S., Antenucci N., Mazzitelli M., Rovira X., Boccella S., Ricciardi F., Liberatore F., Gomez-Santacana X., Imbriglio T., Cannella M., Zussy C., Luongo L., Maione S., Goudet C., Battaglia G., Llebaria A., Nicoletti F., Neugebauer V.A. (2024). “Double-edged” role for type-5 metabotropic glutamate receptors in pain disclosed by light-sensitive drugs.. bioRxiv.

[131] Li J.Q., Chen S.R., Chen H., Cai Y.Q., Pan H.L. (2010). Regulation of increased glutamatergic input to spinal dorsal horn neurons by mGluR5 in diabetic neuropathic pain.. J. Neurochem..

[132] Sotgiu M.L., Bellomi P., Biella G.E.M. (2003). The mGluR5 selective antagonist 6-methyl-2-(phenylethynyl)-pyridine reduces the spinal neuron pain-related activity in mononeuropathic rats.. Neurosci. Lett..

[133] Kartha S., Ghimire P., Winkelstein B.A. (2021). Inhibiting spinal secretory phospholipase A 2 after painful nerve root injury attenuates established pain and spinal neuronal hyperexcitability by altering spinal glutamatergic signaling.. Mol. Pain.

[134] Hsieh M.C., Peng H.Y., Ho Y.C., Lai C.Y., Cheng J.K., Chen G.D., Lin T.B. (2019). Transcription repressor Hes1 contributes to neuropathic pain development by modifying CDK9/RNAPII-dependent spinal mGluR5 transcription.. Int. J. Mol. Sci..

[135] Honda K., Shinoda M., Kondo M., Shimizu K., Yonemoto H., Otsuki K., Akasaka R., Furukawa A., Iwata K. (2017). Sensitization of TRPV1 and TRPA1 *via* peripheral mGluR5 signaling contributes to thermal and mechanical hypersensitivity.. Pain.

[136] Zhang W., Drzymalski D., Sun L., Xu Q., Jiao C., Wang L., Xie S., Qian X., Wu H., Xiao F., Fu F., Feng Y., Chen X. (2018). Involvement of mGluR5 and TRPV1 in visceral nociception in a rat model of uterine cervical distension.. Mol. Pain.

[137] Kim Y.H., Park C.K., Back S.K., Lee C.J., Hwang S.J., Bae Y.C., Na H.S., Kim J.S., Jung S.J., Oh S.B. (2009). Membrane-delimited coupling of TRPV1 and mGluR5 on presynaptic terminals of nociceptive neurons.. J. Neurosci..

[138] Yu J., Du J., Fang J., Liu Y., Xiang X., Liang Y., Shao X., Fang J. (2021). The interaction between P2X3 and TRPV1 in the dorsal root ganglia of adult rats with different pathological pains.. Mol. Pain.

[139] Montana M.C., Gereau R.W. (2011). Metabotropic glutamate receptors as targets for analgesia: Antagonism, activation, and allosteric modulation.. Curr. Pharm. Biotechnol..

[140] Varney M., Gereau R.I.V. (2002). Metabotropic glutamate receptor involvement in models of acute and persistent pain: prospects for the development of novel analgesics.. Curr. Drug Targets CNS Neurol. Disord..

[141] Saab C.Y., Wang J., Gu C., Garner K.N., Al-Chaer E.D. (2006). Microglia: A newly discovered role in visceral hypersensitivity?. Neuron Glia Biol..

[142] D’Antoni S., Berretta A., Bonaccorso C.M., Bruno V., Aronica E., Nicoletti F., Catania M.V. (2008). Metabotropic glutamate receptors in glial cells.. Neurochem. Res..

[143] (2000). Dogrul, Ahmet Peripheral and spinal antihyperalgesic activity of SIB-1757, a metabotropic glutamate receptor (mGLUR5) antagonist, in experimental neuropathic pain in rats.. Neurosci. Lett..

[144] Lindström E., Brusberg M., Hughes P.A., Martin C.M., Brierley S.M., Phillis B.D., Martinsson R., Abrahamsson C., Larsson H., Martinez V., Blackshaw A.L. (2008). Involvement of metabotropic glutamate 5 receptor in visceral pain.. Pain.

[145] Hudson L.J., Bevan S., McNair K., Gentry C., Fox A., Kuhn R., Winter J. (2002). Metabotropic glutamate receptor 5 upregulation in A-fibers after spinal nerve injury: 2-methyl-6-(phenylethynyl)-pyridine (MPEP) reverses the induced thermal hyperalgesia.. J. Neurosci..

[146] Urban M.O., Hama A.T., Bradbury M., Anderson J., Varney M.A., Bristow L. (2003). Role of metabotropic glutamate receptor subtype 5 (mGluR5) in the maintenance of cold hypersensitivity following a peripheral mononeuropathy in the rat.. Neuropharmacology.

[147] Zhu C.Z., Wilson S.G., Mikusa J.P., Wismer C.T., Gauvin D.M., Lynch J.J., Wade C.L., Decker M.W., Honore P. (2004). Assessing the role of metabotropic glutamate receptor 5 in multiple nociceptive modalities.. Eur. J. Pharmacol..

[148] Chung G., Kim C.Y., Yun Y.C., Yoon S.H., Kim M.H., Kim Y.K., Kim S.J. (2017). Upregulation of prefrontal metabotropic glutamate receptor 5 mediates neuropathic pain and negative mood symptoms after spinal nerve injury in rats.. Sci. Rep..

[149] Huang L., Xiao W., Wang Y., Li J., Gong J., Tu E., Long L., Xiao B., Yan X., Wan L. (2024). Metabotropic glutamate receptors (mGluRs) in epileptogenesis: An update on abnormal mGluRs signaling and its therapeutic implications.. Neural Regen. Res..

[150] Niu Y., Zeng X., Zhao L., Zhou Y., Qin G., Zhang D., Fu Q., Zhou J., Chen L. (2020). Metabotropic glutamate receptor 5 regulates synaptic plasticity in a chronic migraine rat model through the PKC/NR2B signal.. J. Headache Pain.

[151] Conn P.J., Pin J.P. (1997). Pharmacology and functions of metabotropic glutamate receptors.. Annu. Rev. Pharmacol. Toxicol..

[152] Xie J.D., Chen S.R., Pan H.L. (2017). Presynaptic mGluR5 receptor controls glutamatergic input through protein kinase C–NMDA receptors in paclitaxel-induced neuropathic pain.. J. Biol. Chem..

[153] Yamakita S., Horii Y., Takemura H., Matsuoka Y., Yamashita A., Yamaguchi Y., Matsuda M., Sawa T., Amaya F. (2018). Synergistic activation of ERK1/2 between A-fiber neurons and glial cells in the DRG contributes to pain hypersensitivity after tissue injury.. Mol. Pain.

[154] Cruz C., Cruz F. (2007). The ERK 1 and 2 pathway in the nervous system: from basic aspects to possible clinical applications in pain and visceral dysfunction.. Curr. Neuropharmacol..

[155] Cavallo D., Landucci E., Gerace E., Lana D., Ugolini F., Henley J.M., Giovannini M.G., Pellegrini-Giampietro D.E. (2020). Neuroprotective effects of mGluR5 activation through the PI3K/Akt pathway and the molecular switch of AMPA receptors.. Neuropharmacology.

[156] He X., Li Y., Deng B., Lin A., Zhang G., Ma M., Wang Y., Yang Y., Kang X. (2022). The PI3K/AKT signalling pathway in inflammation, cell death and glial scar formation after traumatic spinal cord injury: Mechanisms and therapeutic opportunities.. Cell Prolif..

[157] Dolan S., Nolan A.M. (2007). Blockade of metabotropic glutamate receptor 5 activation inhibits mechanical hypersensitivity following abdominal surgery.. Eur. J. Pain.

[158] Boccella S., Marabese I., Iannotta M., Belardo C., Neugebauer V., Mazzitelli M., Pieretti G., Maione S., Palazzo E. (2019). Metabotropic glutamate receptor 5 and 8 modulate the ameliorative effect of ultramicronized palmitoylethanolamide on cognitive decline associated with neuropathic pain.. Int. J. Mol. Sci..

[159] Sheffler D.J., Gregory K.J., Rook J.M., Conn P.J. (2011). Allosteric modulation of metabotropic glutamate receptors.. Adv. Pharmacol..

[160] Gómez-Santacana X., Pittolo S., Rovira X., Lopez M., Zussy C., Dalton J.A.R., Faucherre A., Jopling C., Pin J.P., Ciruela F., Goudet C., Giraldo J., Gorostiza P., Llebaria A. (2017). Illuminating phenylazopyridines to photoswitch metabotropic glutamate receptors: from the flask to the animals.. ACS Cent. Sci..

[161] Fisher K., Fundytus M.E., Cahill C.M., Coderre T.J. (1998). Intrathecal administration of the mGluR compound, (S)-4CPG, attenuates hyperalgesia and allodynia associated with sciatic nerve constriction injury in rats.. Pain.

[162] Walker K., Bowes M., Panesar M., Davis A., Gentry C., Kesingland A., Gasparini F., Spooren W., Stoehr N., Pagano A., Flor P.J., Vranesic I., Lingenhoehl K., Johnson E.C., Varney M., Urban L., Kuhn R. (2001). Metabotropic glutamate receptor subtype 5 (mGlu5) and nociceptive function.. Neuropharmacology.

[163] Montana M.C., Cavallone L.F., Stubbert K.K., Stefanescu A.D., Kharasch E.D., Gereau R.W. (2009). The metabotropic glutamate receptor subtype 5 antagonist fenobam is analgesic and has improved *in vivo* selectivity compared with the prototypical antagonist 2-methyl-6-(phenylethynyl)-pyridine.. J. Pharmacol. Exp. Ther..

[164] Cleva R.M., Watterson L.R., Johnson M.A., Olive M.F. (2012). Differential modulation of thresholds for intracranial self-stimulation by mGlu5 positive and negative allosteric modulators: Implications for effects on drug self-administration.. Front. Pharmacol..

[165] Barnes S.A., Sheffler D.J., Semenova S., Cosford N.D.P., Bespalov A. (2018). Metabotropic glutamate receptor 5 as a target for the treatment of depression and smoking: Robust preclinical data but inconclusive clinical efficacy.. Biol. Psychiatry.

[166] Hu Y., Dong L., Sun B., Guillon M.A., Burbach L.R., Nunn P.A., Liu X., Vilenski O., Ford A.P.D.W., Zhong Y., Rong W. (2009). The role of metabotropic glutamate receptor mGlu5 in control of micturition and bladder nociception.. Neurosci. Lett..

[167] Dalton J.A.R., Pin J.P., Giraldo J. (2017). Analysis of positive and negative allosteric modulation in metabotropic glutamate receptors 4 and 5 with a dual ligand.. Sci. Rep..

[168] Porter R.H.P., Jaeschke G., Spooren W., Ballard T.M., Büttelmann B., Kolczewski S., Peters J.U., Prinssen E., Wichmann J., Vieira E., Mühlemann A., Gatti S., Mutel V., Malherbe P. (2005). Fenobam: A clinically validated nonbenzodiazepine anxiolytic is a potent, selective, and noncompetitive mGlu5 receptor antagonist with inverse agonist activity.. J. Pharmacol. Exp. Ther..

[169] Emmitte K.A. (2017). mGlu5 negative allosteric modulators: A patent review (2013-2016).. Expert Opin. Ther. Pat..

[170] López-Cano M., Font J., Llebaria A., Fernández-Dueñas V., Ciruela F. (2019). Optical modulation of metabotropic glutamate receptor type 5 *in vivo* using a photoactive drug.. Methods Mol. Biol..

[171] Swedberg M.D.B., Raboisson P. (2014). AZD9272 and AZD2066: selective and highly central nervous system penetrant mGluR5 antagonists characterized by their discriminative effects.. J. Pharmacol. Exp. Ther..

[172] (2014). Study to evaluate the analgesic efficacy of 28 days' oral administration of azd2066 compared with placebo in patients with painful diabetic neuropathy.. http://www.ClinicalTrials.gov.

[173] Ai N., Wood R.D., Welsh W.J. (2015). Identification of nitazoxanide as a group i metabotropic glutamate receptor negative modulator for the treatment of neuropathic pain: An in silico drug repositioning study.. Pharm. Res..

[174] Shueb S.S., Erb S.J., Lunzer M.M., Speltz R., Harding-Rose C., Akgün E., Simone D.A., Portoghese P.S. (2019). Targeting MOR-mGluR5 heteromers reduces bone cancer pain by activating MOR and inhibiting mGluR5.. Neuropharmacology.

[175] Aoki T., Narita M., Shibasaki M., Suzuki T. (2004). Metabotropic glutamate receptor 5 localized in the limbic forebrain is critical for the development of morphine‐induced rewarding effect in mice.. Eur. J. Neurosci..

[176] Gabra B.H., Smith F.L., Navarro H.A., Carroll F.I., Dewey W.L. (2008). mGluR5 antagonists that block calcium mobilization *in vitro* also reverse (S)-3,5-DHPG-induced hyperalgesia and morphine antinociceptive tolerance *in vivo*.. Brain Res..

[177] Akgün E., Javed M.I., Lunzer M.M., Smeester B.A., Beitz A.J., Portoghese P.S. (2013). Ligands that interact with putative MOR-mGluR5 heteromer in mice with inflammatory pain produce potent antinociception.. Proc. Natl. Acad. Sci. USA.

